# OmicStudio: A composable bioinformatics cloud platform with real‐time feedback that can generate high‐quality graphs for publication

**DOI:** 10.1002/imt2.85

**Published:** 2023-02-06

**Authors:** Fengye Lyu, Feiran Han, Changli Ge, Weikang Mao, Li Chen, Huipeng Hu, Guoguo Chen, Qiulei Lang, Chao Fang

**Affiliations:** ^1^ Operation Department LC‐Bio Technology Co., Ltd. Hangzhou China

## Abstract

OmicStudio focuses on speed, quality together with flexibility. Generally, OmicStudio can not only meet the users' demand of ordinary bioinformatics data analysis, statistics, and visualization, but also provides them freedom of data mining beyond developer's framework. Additionally, unlimited to developer's aesthetics, users can get more elegant graphs through customizing. Available online https://www.omicstudio.cn.

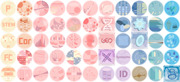

In the past decade, an increasing number of cloud‐based bioinformatics platforms were spring up, such as Qiita [1], EasyMAP [2], ImageGP [3], MG‐RAST [4], gcMeta [5], ETCM [6], Sangerbox [7], antiSMASH [8], EVenn [9], and Majorbio Cloud [10]. These platforms greatly facilitated the biological, medicine and metagenomics research, and provided new vision into big data. Some software deploy various independent tools [11–16], while the others provide complete pipeline [17–21]. The main highlight of these cloud platforms is to convert complicated coding works into easy‐used web‐based tools [22]. With the assumption that all users already have enough knowledge about bioinformatics before operation, these tools mainly focus on simplifying operation procedure. But the unnoticed truth is that users are also learning while operating, who at the same time expect to get graphs for publication through simple operation.

Here, we display the overall framework as well as each independent module of OmicStudio Cloud Platform (https://www.omicstudio.cn). OmicStudio is a one‐stop online analysis platform providing high‐throughput omics data analysis, as well as an exploratory platform for bioinformatics research. OmicStudio can obtain results rapidly, generate high‐quality graphs for publication, and connect with downstream analysis module automatically. It focuses on speed, quality together with flexibility. Generally, OmicStudio can not only meet the users' demand of ordinary bioinformatics data analysis, statistics, and visualization but also provides them freedom of data mining beyond developer's framework. Additionally, unlimited to developer's aesthetics, users can get more elegant graphs through customizing.

In terms of website framework, OmicStudio has common configuration that all bioinformatics cloud platform may have, such as Cloud Tools, Cloud Analysis, Omics Courses and Study Materials Center, providing entire bioinformatics service for users.

Since its establishment in early 2019, OmicStudio already attracted over 30,000 users, helped whom publish more than 600 research papers (search “OmicStudio” in Google scholar on January 1, 2023), involving various “‐omics” fields. An easy‐to‐use platform which can generate high‐quality results is obviously able to make great contribution to scientific research.

## OMICSTUDIO OVERVIEW

OmicStudio consists of six modules, named Cloud Tools, Cloud Analysis, Cloud Classroom, Study Materials Center, User's Paper Collection, and User Center. Cloud Tools and Cloud Analysis are the main part of the function for bioinformatics analysis. Cloud Classroom and Study Materials Center help users understand and learn bioinformatics. User's Paper Collection collects the papers published in cooperation with LCBIO and users can take them as reference. User Center is an integrated management module for user's project information. The core functions of the cloud platform are cloud tools and cloud analytics. The main difference is that cloud tools focus on short, quick analysis, while cloud analytics can support time‐consuming analysis and have a project management back office. The two modules are distinguished by the nature of the analysis: the analysis with large amount of data and complex calculation needs to be able to run in the background so that users can leave the web page and do their own thing; simple analytics require instant results and can be personalized immediately. Based on this consideration, the module design can allow users to obtain the most comfortable experience in different application scenarios. Other modules serve these two core modules. Cloud Classroom and Study Materials Center provide the basic knowledge of run bioinformatics analysis in the form of video and text respectively. User Center manages the analysis results, and User's Paper Collection provide new users with reference for writing articles. The innovativeness and character of OmicStudio major function will be clarified later.

**Figure 1:**
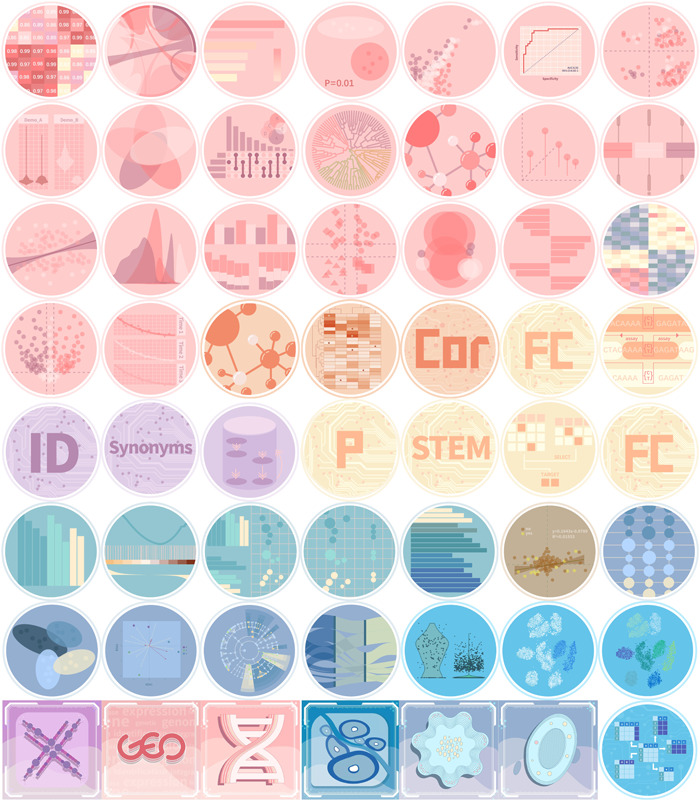
Overview tools in OmicStudio. Colors indicate different omics. Pink, General Omics; blue, Single Cell; green, Enrichment Analysis; yellow, Common Analysis; purple, Databases; orange, correlation; bluish violet, microbiome; dark blue, tumor WES. Shape of the icons indicates tool category: circle—Tools, which response immediately; square—Analysis, which runs as a workflow and takes relatively long time.

In terms of type of omics and application scenarios, Cloud Tools are classified as: General Omics, Single Cell, Enrichment Analysis, Common Analysis, Databases, and so on (Figure 1). The summary of the useful tool in OmicStudio s are showed in Figure 1.

## OMICSTUDIO FEATURES

### Effect of parameter adjustment with real‐time feedback

“Set the parameter and start to analyze,” ordinary online bioinformatics tools usually follow this principle. First, users are required to finish the parameter adjustment with unpredictable result, which is with great difficulty. The process of learning that most of us are used to be the “adjust—view the results—adjust again—view the results again” loop. After parameter adjustment and analysis, users need to view the results in backstage. If readjustment is needed, users need to click back to the previous web page, readjust the settings, and view the results again. This is a tedious process with a lot of repetition. Second, for those unfamiliar with bioinformatics, the corresponding relationship between parameter and result is unclear. Consequently, they cannot acquire the desired result through adjusting parameters. Either they are required to spend much time learning the meaning of parameter, which has nothing to do with their goal, or they must do repetitive work repeatedly. Thirdly, there is no uniform standard for development of bioinformatics website. As a result, users must spend time learning how to use these various online tools. Usually, the function of a bioinformatics tool may be simplified to improve user experience, thus users could get desired results without paying too much time cost. But this simplification will not only limit users' cognition (they don't know how much key information is hidden from their simple operation) but also restrict these online tools to becoming multifunctional platforms, which can provide more personalized bioinformatics tools to meet the demand of more advanced scientific research.

OmicStudio Cloud Platform focuses on real‐time feedback. The results of parameter adjustment can be visualized immediately just on the current web page, which greatly reduces users' time cost of debugging. Furthermore, OmicStudio helps users understand the corresponding relationship between parameters and results in an interesting way, turning tedious data analysis into a game. In this game, users can fully understand bioinformatics and how the tools on this platform work. Real‐time feedback makes the influence of parameter more comprehensible without knowing its connotation, and then helps data users understand the meaning of parameter through its influence. OmicStudio overcomes the difficulty for users in understanding the esoteric principles of bioinformatics and tools operation, which is a great breakthrough in development of bioinformatics platform.

### Providing default parameter, one‐click analyze

As mentioned above, the more parameters result in the higher cost of learning. To solve this problem, OmicStudio provides default parameters. In the most convenient way, users only need to upload the documents, wait for the refreshment of the image on the web page (it usually takes just a few seconds), and then download. Despite great number of parameters, each of them has a default value, with which OmicStudio can immediately generate a graph for publication. If users just want to have a glance at their data quality (e.g., using principal component analysis to examine the sample repetition, or using correlation heatmap to check the relevance of data), they can directly download and use, which can be done within 1 min. Default parameters are parameters most frequently used, which are reliable in most cases, and will not be the barrier to choose.

### High‐quality and free adjustable analysis results

Ordinary bioinformatics platforms focus only on the analysis procedure. As a result, for publication purposes, the generated graph is usually needed to be redrawn and readjusted. OmicStudio also provides the adjustment module for drawing graphs. The default style of graphs is well designed and adjustable. Users can adjust the themes, color schemes, fonts, titles, and so on. If OmicStudio cannot meet all the needs, it also provides a further solution: downloadable figures in PowerPoint format. The figure generated from OmicStudio can be split into elements in PowerPoint, each of which can be adjusted independently. The function provided by PowerPoint can all be applied to the adjustment of graphs. It has the same function with Adobe Illustrator, while with lower cost of learning.

### Associate with downstream analysis module freely

**Figure 2:**
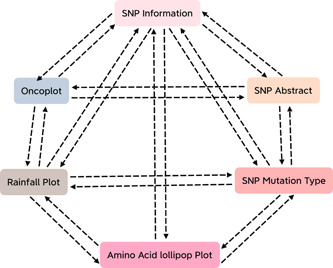
Flowchart illustrating the relationship between cloud tools

For scientific research, besides the free adjustable parameters, the further demand is to freely choose what to do in the downstream analysis. Although ordinary bioinformatics cloud platforms can provide easy‐to‐use analysis procedure, they may limit users to freely choosing what to analyze. The analysis modules of OmicStudio are independent while correlated. As shown below, on the page of one kind of cloud tool, there is a series of related cloud tools displayed on the right sidebar, and users can click it to jump to the page of analysis (analysis can be continued based on current results, and there is no necessary to upload any document). The advantage of it is that users can freely choose what to analyze next, as well as decide when to stop. Figure 2 displays the correlation among cloud tools for Tumor WES. Similar series of modules includes Correlation Analysis, Enrichment Analysis, and so on.

### High‐quality graph for publication

**Figure 3:**
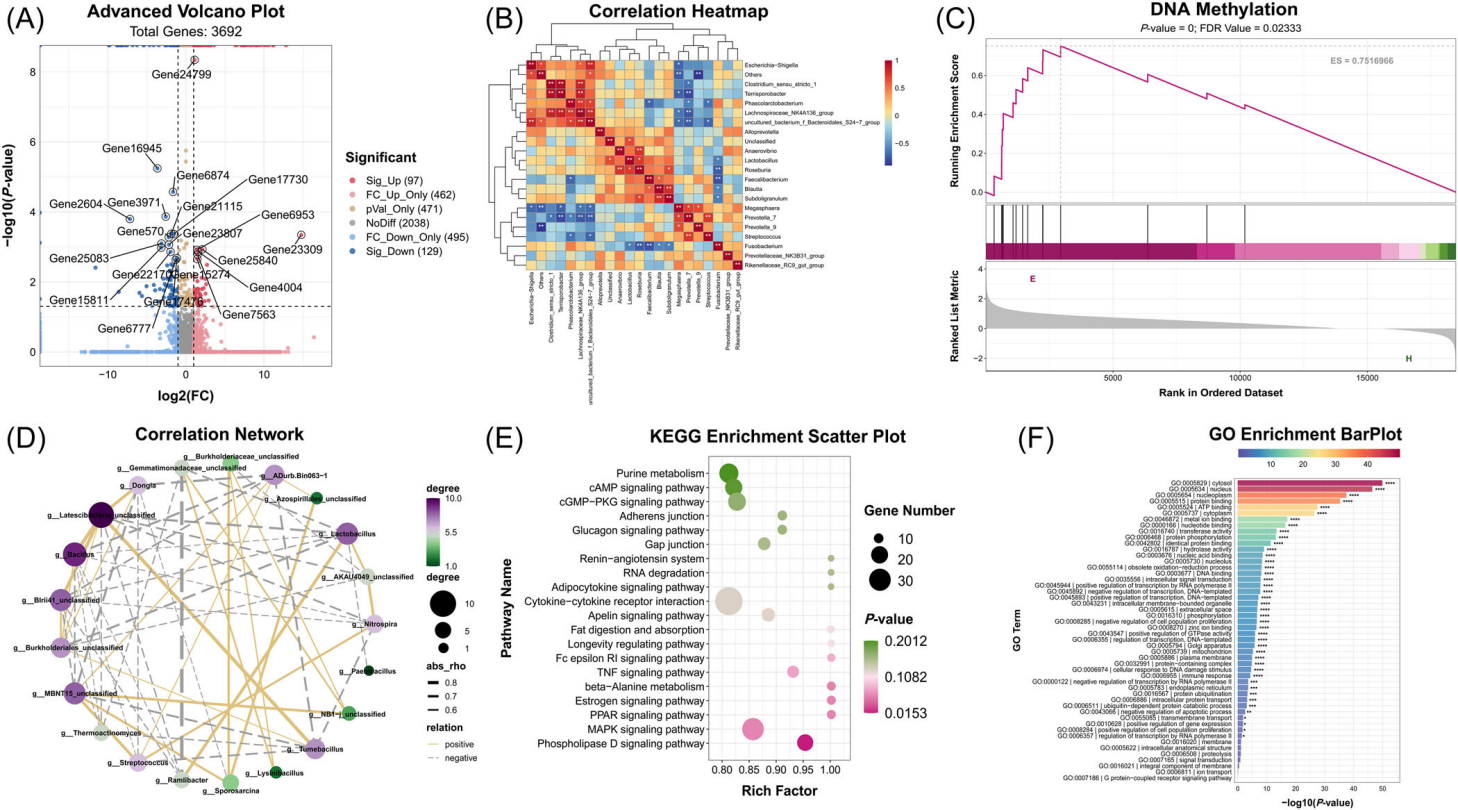
Examples of graphs: (A) volcano plot, (B) correlation heatmap, (C) GSEA Enrichment, (D) correlation network, (E) KEGG enrichment scatter plot, and (F) Classic Enrichment.

Graphs generated by OmicStudio are famous for its beautiful design. Typical examples are shown in Figure 3, which were all downloaded directly from OmicStudio without any extra adjustment. Graphs from OmicStudio are so well designed that they can still meet the standard of aesthetic even after some customized adjustment. In addition, OmicStudio Cloud Platform supports graphic parameter adjustment, such as fonts, color schemes, themes, and axis adjustment. It's convenient for users to customize the graph according to their demand.

### Automatically save the analysis parameter, convenient to backtrack

Ordinary bioinformatics cloud tools save the analysis parameter in background, which is invisible to users. When writing a research paper, it is difficult for users to get the value of parameter from the bioinformatics software.

OmicStudio, on the contrary, provides users parameters along with analysis results. Whether how much time goes by, whether how many versions have been updated, users can always find the parameters in their analysis. Downloadable records of analysis include: reference, analysis method, version of software, analysis parameter, basic information of analysis (when and which tools are used in the platform), citation. This series of information establish a foundation for users' writing.

## EXTRA RESOURCES FOR USERS

Cloud Classroom module includes 9 themes and nearly 100 courses. It's convenient to focus on any course based on demand. The themes include bioinformatics, omics science knowledge and literature interpretation, aiming to meet all the users' demand from understanding of biology to the learning of operation.

User's Paper Collection module collects hundreds of research articles published by OmicStudio users, providing name of journal, year of publication, impact factor, source of sample, method used and other information. Users can search, preview, and download pdf version of paper. It's convenient for users to acquire knowledge in interested field, and reproduce the analysis result by OmicStudio.

OmicStudio focuses on making scientific research more easily. Since October 2019, the services content of the platform has been upgraded and iterated over 1000 times. There are already more than 30,000 scientific research users. In the past 3 years, 669 research articles cited OmicStudio (Google Scholar, by January 1, 2022).

## AUTHOR CONTRIBUTIONS

Fengye Lyu designed the platform and idea. Fengye Lyu and Feiran Han wrote the manuscript. Feiran Han was responsible for editing and revising the manuscript. All authors contributed to the development of OmicStudio Platform.

## CONFLICTS OF INTEREST STATEMENT

Qiulei Lang is the Shareholders of LC‐Bio Technology Co. The other authors are employees of LC‐Bio.

## Data Availability

There is no data available.
